# The Predictive Capacity of the Buffalo Concussion Treadmill Test After Sport-Related Concussion in Adolescents

**DOI:** 10.3389/fneur.2019.00395

**Published:** 2019-04-24

**Authors:** Mohammad N. Haider, John J. Leddy, Charles G. Wilber, Kaitlin B. Viera, Itai Bezherano, Kimberly J. Wilkins, Jeffrey C. Miecznikowski, Barry S. Willer

**Affiliations:** ^1^UBMD Orthopedics and Sports Medicine, Jacobs School of Medicine and Biomedical Sciences, State University of New York, Buffalo, NY, United States; ^2^Department of Neuroscience, Jacobs School of Medicine and Biomedical Sciences, State University of New York, Buffalo, NY, United States; ^3^Department of Family Medicine, Jacobs School of Medicine and Biomedical Sciences, State University of New York, Buffalo, NY, United States; ^4^Department of Biostatistics, School of Public Health and Health Professions, State University of New York, Buffalo, NY, United States; ^5^Department of Psychiatry, Jacobs School of Medicine and Biomedical Sciences, State University of New York at Buffalo, Buffalo, NY, United States

**Keywords:** Buffalo Concussion Treadmill Test, sport-related concussion, adolescent, post-concussion syndrome, exercise intolerance

## Abstract

The Buffalo Concussion Treadmill Test (BCTT) identifies the heart rate threshold (HRt) of exercise tolerance in concussed patients. A previous study found that an absolute HRt of < 135 bpm was associated with prolonged recovery (>30 days) from sport-related concussion (SRC). In this study, we assessed the relationship of ΔHR (difference between resting HR and HRt) and recovery from SRC. Using a retrospective cohort design, we compared acutely (<10 days since injury) concussed adolescents who were prescribed either (1) relative rest (RG, *n* = 27, 15.2 ± 1 years, 33% female, median 17 days to recovery, ΔHR = 69.6 ± 28 bpm), (2) a placebo-stretching program (PG, *n* = 51, 15.4 ± 2 years, 49% female, median 17 days to recovery, ΔHR = 60.9 ± 22 bpm), or (3) sub-threshold aerobic exercise (AG, *n* = 52, 15.3 ± 2 years, 46% female, median 13 days to recovery, ΔHR = 62.4 ± 26 bpm). Linear regression showed that ΔHR significantly correlated with duration of clinical recovery for RG (*p* = 0.012, *R*^2^ = 0.228) and PG (*p* = 0.011, *R*^2^ = 0.126) but not for AG (*p* = 0.084, *R*^2^ = 0.059). ΔHR values were significantly lower in participants with prolonged recovery (>30 days) in RG (*p* = 0.01) and PG (*p* = 0.04). A ΔHR of ≤50 bpm on the BCTT is 73% sensitive and 78% specific for predicting prolonged recovery in concussed adolescents who were prescribed the current standard of care (i.e., cognitive and physical rest).

## Introduction

Sport-related concussion (SRC), a type of mild traumatic brain injury (mTBI), is a significant public health concern (1, 2). Concussion is defined as reversible neurological dysfunction in the absence of gross brain lesions, caused by either by a direct blow to the head, neck, or elsewhere on the body with an impulsive force transmitted to the head (3, 4). SRC presents with a variety of somatic, cognitive, and affective symptoms (5). Symptom-limited exercise intolerance, i.e., the inability to exercise to the level predicted for one's age and fitness because of symptom exacerbation, helps to define physiological dysfunction after SRC (6). The degree of exercise intolerance within the first week after SRC is a strong indicator of the severity of SRC (7, 8). The cause for exercise intolerance after concussion is not fully understood but may be related to damage to the brainstem that uncouples the autonomic nervous system (ANS) from the cardiovascular system (9, 10). It is theorized that abnormal ANS function alters cerebral blood flow (CBF) regulation during exercise that produces symptoms of headache and dizziness to limit exercise duration (11, 12). Most patients recover from SRC in 7 to 10 days but up to 30% take longer to recover (13, 14). If symptoms persist for more than 2 weeks in adults and for more than 1 month in adolescents, then they are described as having Persistent Post-concussive symptoms (PPCS) (15).

The Buffalo Concussion Treadmill Test (BCTT) (16) is a validated test to measure the amount of aerobic exercise that is safe to perform, even in the acute phase after concussion (17, 18). The heart rate (HR) achieved at symptom exacerbation on the BCTT is called the heart rate threshold (HRt). In a previous randomized controlled trial in acutely concussed adolescents (17), a HRt < 135 bpm was significantly associated with recovery of > 21 days. An absolute HRt cut-off value is, however, not appropriate for everyone due to the large variation in resting HR, which is dependent on multiple factors, including cardiovascular fitness (19). In an attempt to develop a predictor better suited to individual differences in fitness, the purpose of this study was to determine whether the difference between resting HR and HRt (the ΔHR) correlated with duration of clinical recovery. Since the standard of care for SRC is changing to a more active approach (20, 21), we included a prior cohort of 27 adolescents prescribed rest in addition to 2 groups of acutely concussed adolescents who were prescribed either a placebo-stretching program or sub-symptom threshold aerobic exercise. Adolescents were studied because they are predominantly concussed in sport (22) and take the longest to recover (23). We hypothesized that the ΔHR on the BCTT within 10 days of injury would correlate with duration of clinical recovery and that participants who developed PPCS would have significantly lower ΔHR than participants who did not. Our secondary aim was to establish a ΔHR value that differentiated between normal and prolonged recovery.

## Materials and Methods

Data from two published randomized controlled trials were used for the current study, the first (17) recruited between March 2013 and February 2015 (clinicaltrials.gov: NCT02714192) and the second (24) recruited between September 2015 and June 2018 (clinicaltrials.gov: NCT02710123). The University at Buffalo Institutional Review Board approved both studies.

### Study Design

Experienced sports medicine physicians evaluated adolescent athletes seen at the University Concussion Management Clinics within 10 days of injury. If eligible for the study, a research assistant explained the study and obtained consent on the same day. Parental consent was obtained for all minors. Sports medicine physicians diagnosed concussion based on a thorough history (including cognitive evaluation and concussion symptom questionnaire) (25) and a standardized physical examination (26). Participants then performed the BCTT to assess degree of exercise tolerance. All participants reported symptoms online daily on a password-protected website between 7 and 10 p.m. using the Post-Concussion Symptom Scale (PCSS) (27) until they were cleared for return-to-play (RTP) or for up to 4 weeks, whichever came first. For those participants who recovered after 4 weeks, the date of recovery was retrospectively determined by electronic medical records. Recovery was defined as symptom resolution to baseline, confirmed by a physician performed physical examination, and the ability to exercise to exhaustion without exacerbation of symptoms on the BCTT (28).

### Intervention

#### Rest Group

Participants in the Rest Group (RG) were prescribed cognitive and physical rest according to the previous standard of care (29). They were told that rest was recommended to give their concussed brain a chance to heal. Rest was described as not participating in any sports or other forms of exercise, including gym class. They were told to limit activities that could exacerbate symptoms such as watching TV or using their phones. Participants were seen every week and the same advice was given until clinical recovery. Participants were referred for cervical or vestibular therapy as needed if they did not recover by 30 days since injury.

#### Placebo Group

Participants in the Placebo Group (PG) were prescribed cognitive rest and were instructed to perform a standardized combination of light stretches and breathing exercises that would not elevate HR. They were given a Polar HR monitor (Model #FIT N2965, Kempele, Finland) to monitor their HR while stretching. They were instructed to not participate in sports or other physical activities that would raise their HR. They were also told to limit activities that could exacerbate symptoms such as excessive use of computer screens or using their phones. Participants were seen every week to perform the BCTT and a new set of stretching exercises was given until recovery. Participants were referred for cervical or vestibular therapy as needed if they did not recover by 30 days since injury.

#### Aerobic Group

Participants in the Aerobic Group (AG) were instructed to perform aerobic exercise (i.e., walking, jogging, or stationary cycling) at 80% of the HRt achieved on the BCTT for 20 min a day. They were given a Polar HR monitor (Model #FIT N2965, Kempele, Finland) to exercise according to their HR prescription. They were instructed to not participate in sports or any forms of physical exercise apart from the prescribed 20-min of aerobic exercise. If the participants felt symptomatic while exercising at home, then they were instructed to stop and rest, and continue the following day. Participants were seen every week to perform the BCTT and a new HR prescription was given until recovery. Participants were referred for cervical or vestibular therapy as needed if they did not recover by 30 days since injury. Further details on PG and AG exercise prescriptions are provided in a recent study (24).

### Participants

Male and female adolescent athletes (aged 13–18 years) presenting within 10 days of SRC were diagnosed with concussion according to international Concussion In Sport Group (CISG) criteria (20). Participants were excluded because of (1) evidence of focal neurological deficit; (2) inability to safely walk on a treadmill due to orthopedic injury or significant vestibular dysfunction; (3) increased cardiac risk according to American College of Sports Medicine criteria (30); (4) history of moderate or severe TBI defined as brain injury with a Glasgow Coma Scale score of 12 or less; (5) current diagnosis of and treatment for ADHD, learning disorder, depression, anxiety, or history of more than 3 prior concussions (because these factors are associated with delayed recovery) (31); (6) sustaining another head injury during the research period before recovery; (7) symptom severity score of <5 on initial visit symptom questionnaire; and (8) limited English proficiency.

### BCTT and Calculation of ΔHR

Before beginning the BCTT, the participant rated his/her symptoms on a Visual Analog Scale (VAS, 0–10) (32) and resting HR was measured in a seated position after 2 min of rest by Polar HR monitor (Model #FIT N2965, Kempele, Finland). The participant then walked on a level treadmill at 3.2 mph (3.6 mph in participants 5′10″ and above) at 0 degree incline. The incline was increased by 1 degree after each minute for the first 15 min and then the speed was increased by 0.4 mph every minute thereafter. HR, VAS, and Borg Rating of Perceived Exertion (RPE) (33) were recorded each minute until symptom exacerbation or voluntary exhaustion. This was followed by a 2-min cool down at 2 mph unless the participant opted out of it. Symptom exacerbation was defined as an increase of 3 points or more from the pre-exercise VAS value (a point or more for an increase in symptoms and a point for appearance of a new symptom). Voluntary exhaustion was defined by a report of 17 or more on the RPE scale. Participants were instructed to report symptoms and to not “push through” them. The examiner also observed for visible signs of distress, which could prompt test cessation. If the participant was unable to reach age-appropriate exercise tolerance, the HR at exercise cessation was recorded as the HRt. ΔHR was calculated as the difference between resting HR and HRt.

### Statistical Analysis

ANOVA was used to assess for group-wise differences in age, days since injury to initial visit, initial PCSS score, resting HR, HRt, and ΔHR. Non-parametric tests of medians was used to compare the non-normally distributed variable duration of clinical recovery. Chi-squared tests were used to assess group-wise differences in gender, history of concussions, and incidence of PPCS. A nonparametric *t*-test assessed differences in ΔHR between normal recovery and PPCS subjects within each group. Linear regression assessed the association between ΔHR and days to recovery within each group. After analysis, AG's ΔHR did not correlate with duration to recovery and only had 2 participants out of 52 with PPCS so they were not included in subsequent analyses. Subjects in each group were dichotomized into those who had normal recovery (≤30 days recovery) and those who developed PPCS (>30 days recovery) and a Receiver Operating Characteristic (ROC) curve analysis using ΔHR as a predictor was performed using participants from RG and PG only. A *p* ≤ 0.05 was considered significant. No power analysis was done. All data analyses were performed using SPSS 24 (Armonk, NY).

## Results

A total of 161 eligible adolescents came to the Concussion Clinic within 10 days of concussive head injury. Fifteen participants were either not interested or did not have time to participate. After randomization, 16 participants were lost to follow-up because they did not return to the clinic or did not complete at least 60% of the daily symptom reports. They were excluded because we could not determine their date of recovery. There were no statistically significant differences in age, height, weight, sex, or initial BCTT results between those that were included in the study and those that dropped out. Hence, 27 participants made up RG, 51 participants made up PG and 52 participants made up AG. Demographic data for each group are presented in Table 1.

**Table 1:** Participant demographics.

	**Rest Group**	**Placebo Group**	**Aerobic Group**	***p*-value**
	**(*n* = 27)**	**(*n* = 51)**	**(*n* = 52)**	
Age (years)	15.2 ± 1.4	15.4 ± 1.7	15.3 ± 1.6	0.81
Sex	33% female	49% female	47% female	0.40
**Previous Concussions**				0.47
0	18	30	26	
1	8	11	16	
2	1	8	9	
3	0	2	1	
Days since injury	4.2 ± 2	4.8 ± 2	4.9 ± 2	0.31
Weight (kg)	63.3 ± 11.6	66.2 ± 12.8	64.2 ± 13.0	0.590
Height (m)	1.68 ± 0.09	1.67 ± 0.10	1.69 ± 0.11	0.579
Symptom severity (PCSS, max = 132)^a^	35.8 ± 23.0	33.5 ± 19.7	30.8 ± 16.5	0.52

*Data are presented as mean ± standard deviations*.

a *Post-Concussion Symptom Scale*.

Table 2 shows the results of the BCTT performed at initial clinic visit (<10 days since injury). There were no significant differences in the initial visit BCTT results between the three groups. Linear regression showed that ΔHR was significantly correlated with duration of clinical recovery for RG (*p* = 0.012, *R*^2^ = 0.228) and PG (*p* = 0.011, *R*^2^ = 0.126) but not for AG (*p* = 0.084, *R*^2^ = 0.059).

**Table 2:** Buffalo concussion treadmill test results.

	**Rest Group**	**Placebo Group**	**Aerobic Group**	***p*-value**
	**(*n*= 27)**	**(*n* = 51)**	**(*n* = 52)**	
Resting HR^a^ (bpm)	75.4 ± 12.1	74.9 ± 12.4	74.5 ± 12.7	0.96
HRt^b^ (bpm)	145.0 ± 24.4	135.8 ± 21.8	136.9 ± 26.2	0.26
ΔHR^c^ (bpm)	69.6 ± 28.5	60.9 ± 21.8	62.4 ± 25.6	0.33
Symptom Exacerbation	81.5% (*n* = 22)	96.1% (*n* = 49)	90.4% (*n* = 47)	0.11
Median Duration of Recovery (IQR^d^)	17 (9.25–23.25)	17 (13–23)	13 (10–18.75)	0.04
Developed PPCS^e^	14.8% (*n* = 4)	13.7% (*n* = 7)	3.8% (*n* = 2)	0.19

a *Heart rate*.

b *Heart rate threshold*.

c *Heart rate difference*.

d *Inter quartile range*.

e *Persistent post-concussive symptoms*.

Table 3 shows mean ΔHR for participants who developed PPCS for each group. Non-parametric *t*-test showed that mean ΔHR for patients who developed PPCS was significantly lower in RG and PG. No analysis between ΔHR and PPCS was performed for AG because there were only 2 participants in the delayed recovery group and ΔHR was not significantly associated with duration of clinical recovery in that group.

**Table 3:** Mean heart rate of participants who developed persistent post-concussive symptoms.

**Rest Group (*****n*** **=** **27)**			***p*-value**
	Developed PPCS (*n* = 4)	Normal Recovery (*n* = 23)	
Mean ΔHR^a^ (bpm)	35.25 ± 9.5	75.57 ± 26.4	**0.01**
**Placebo Group (*****n*** **=** **51)**			
	Developed PPCS (*n* = 7)	Normal Recovery (*n* = 44)	
Mean ΔHR (bpm)	43.43 ± 20.5	63.73 ± 20.9	**0.04**

a *Heart rate difference, PPCS > 30 days recovery duration, normal recovery ≤ 30 days recovery duration*.

**Figure 1:**
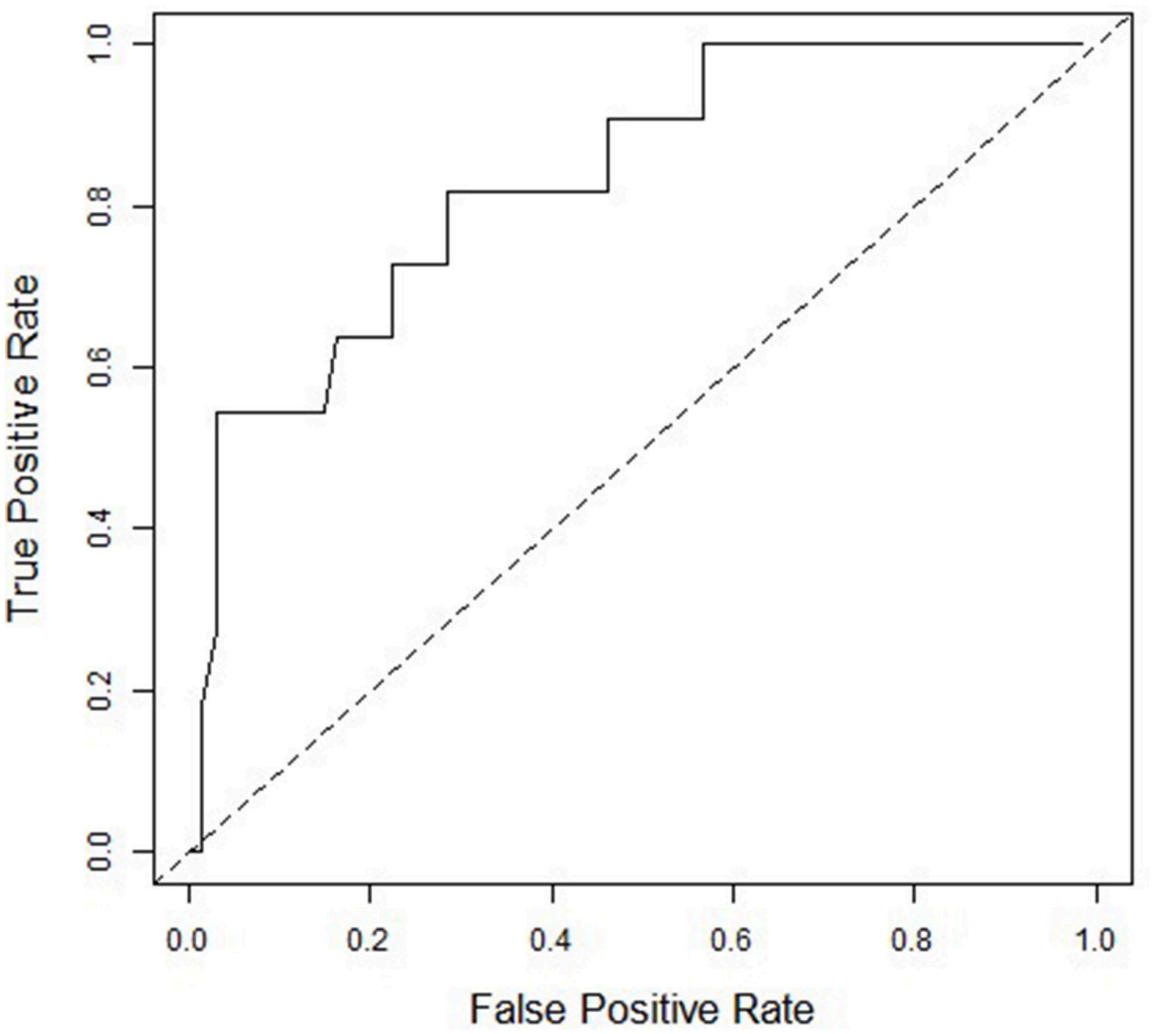
Receiver Operating Characteristics (ROC) curve for ΔHR and Persistent Post-Concussive Symptoms (PPCS) for Rest Group and Placebo Group combined (*n* = 78). An ROC analysis of the placebo and rest group showing the ability of ΔHR to predict PPCS. Area under the curve is 0.81. At the ΔHR of <50 bpm to declare PPCS, sensitivity = 73% and 1-specificity = 22%.

Figure 1 presents the ROC curve of ΔHR and PPCS of RG and PG combined (67 normal recovery vs. 11 PPCS). The ΔHR of ≤50 bpm was 73% sensitive (8/11) and 78% specific (52/67) for identifying adolescents who experienced a delayed recovery. AG were not included because ΔHR did not correlate with duration of clinical recovery.

## Discussion

We found that the ΔHR correlated with duration of clinical recovery in participants who were prescribed relative rest or a placebo-stretching program but not in participants who were prescribed sub-threshold aerobic exercise. A ΔHR value of ≤50 bpm was 73% sensitive and 78% specific for identifying adolescents who experienced delayed recovery. Other values would have greater specificity but at the cost of reduced sensitivity. Greater sensitivity is more useful to clinicians who are trying to identify those patients who are more likely to have significant difficulty with schoolwork, social relationships and sports team participation after SRC. Normal resting HR values have a wide distribution (between 60 and 100 bpm), which is dependent on modifiable and non-modifiable factors such as age and cardiovascular fitness (34). Our previous study suggested that an absolute cut-off HRt value of more than 135 bpm was associated with normal recovery after SRC vs. those with a HRt < 135 bpm who were much more likely to experience a delayed recovery. We were, however, limited in our ability to accurately predict delayed recovery since only 4 patients (out of 27) developed PPCS. In addition, the previous study used a definition of >21 days to classify delayed recovery. The change in definition of delayed recovery is inform the newer CISG definition (20, 29). On retrospective analysis, using >30 days did not affect the results of the previous study. Furthermore, an absolute cut-off value does not account for differences in resting HR. For example, a patient with a resting HR of 60 bpm who attains a HRt of 140 bpm on the BCTT likely has a better prognosis than one who reaches that level from a resting HR of 90 bpm.

Symptom severity in the acute and sub-acute period, as assessed by concussion-specific symptom checklists, is considered to be the most accurate predictor of recovery duration after SRC (23). One reason for this is that almost all studies define recovery as return to a normal or baseline level of symptoms (28). While symptom reports are essential for the management of SRC, there are issues with clinicians relying on subjective reporting alone to establish recovery from SRC. Athletes, for example, are known to under-report symptoms to avoid missing their sport (35) whereas persons with secondary gain issues have been known to over-report symptoms (36). Symptom questionnaires may cause reporting bias and encourage the over-endorsement of symptoms, which may not have been reported on free recall (37, 38). For these reasons, researchers are searching for more objective measures of concussion/mTBI severity and predictors of duration of clinical recovery (39). The ΔHR measure on an exercise test performed early after concussion may be a clinically reasonable physiological biomarker of concussion severity because it requires readily available equipment (unlike advanced imaging), is non-invasive (unlike blood tests), is relatively easy to perform, and has been shown to be safe to perform as soon as 2 days after SRC (17).

The ΔHR of the group that performed a placebo-like stretching program was significantly correlated with duration of clinical recovery, which is similar to the group prescribed relative rest in our prior study (17). This was not unexpected because the placebo was designed to mimic relative rest, which is the standard of care (29). RG and PG had almost identical recovery times (17 days) and incidence of PPCS (15 and 14%, respectively) so we are confident that our placebo-like stretching program effectively mimicked rest. Our hypothesis that the ΔHR would be a prognostic indicator of recovery time irrespective of treatment was not confirmed. We suspect that this is because prescribed sub-threshold aerobic exercise treatment reduced recovery time such that a pre-intervention predictor variable of exercise intolerance was no longer valid. There is emerging research to suggest that light to moderate physical activity that does not exacerbate symptoms is beneficial for patients with concussion and reduces recovery time (18, 38, 40–42). In a recent randomized placebo-controlled trial, (24) we showed that sub-threshold aerobic exercise prescribed in the sub-acute phase after SRC safely and significantly reduced recovery time from a median of 17 to 13 days. The mechanism for the beneficial effect of sub-symptom threshold exercise on concussion is not completely understood but may include salutary effects on autonomic nervous system function, control of cerebral blood flow, cognition, mood, sleep, and upon neuroplasticity via increasing levels of brain-derived neurotrophic factor (43–46).

### Limitations

There are several limitations to our study. HR varies because it is influenced by fitness level, emotional state, amount and time since food intake, and time of day. We did not control for all of these variables. This, however, increases the external validity of the study. We studied only adolescents so the results cannot be generalized to younger children or adults. As with any clinical test, results of the BCTT are dependent on the clinician performing the test. The BCTT can be performed by anyone who is trained to perform exertion testing. In our setting (a university concussion management clinic), the BCTT is usually performed by physical therapists, athletic trainers, or exercise science students. RG participants completed daily symptom reports whereas PG and AG completed daily symptom reports plus self-reported compliance with the prescribed intervention. Compliance reported by AG and PG was 83 and 86%, respectively, but we cannot be sure if this is accurate, and we cannot be sure if participants adhered to the recommendation to not perform sports or other physical exercise during intervention. Lastly, only two participants in the AG group experienced delayed recovery so we were not able to perform a statistical analysis on participants who had normal recovery vs. those who did not. Therefore, the ROC analysis for ΔHR is applicable only to concussed adolescents who were been prescribed the current standard of care (i.e., physical and cognitive rest) and not to those prescribed sub-threshold aerobic exercise.

## Conclusion

This study found that the ΔHR (HRt minus resting HR) correlated with duration of clinical recovery in participants who were prescribed relative rest or a placebo-stretching program but not for participants prescribed sub-threshold aerobic exercise. A ΔHR of ≤50 bpm on the BCTT was 73% sensitive and 78% specific for predicting delayed recovery in concussed adolescents prescribed the current standard of care (i.e., cognitive and physical rest). This has implications for planning team and school activities in adolescents who sustain SRC.

## Ethics Statement

This study was carried out in accordance with the recommendations of Institutional Review Board, University at Buffalo, SUNY with written informed consent from all subjects. All subjects gave written informed consent in accordance with the Declaration of Helsinki. The protocol was approved by the University at Buffalo, SUNY.

## Author Contributions

MH, JL, and BW contributed to the inception and design of the paper and writing of the manuscript. KW, CW, IB, and KV contributed to patient recruitment and data collection. JM contributed to the statistical analysis and research design. All authors approved the final version of the manuscript.

### Conflict of Interest Statement

The authors declare that the research was conducted in the absence of any commercial or financial relationships that could be construed as a potential conflict of interest.
